# Case Report: Multidisciplinary management of a rare labial–palatal dual developmental groove with malocclusion: a 4-year follow-up

**DOI:** 10.3389/froh.2025.1705402

**Published:** 2025-11-20

**Authors:** Zhaowei Tai, Huxiao Li, Shuang Li, Jian Wang

**Affiliations:** 1 Department of General Dentistry, Shanghai Ninth People's Hospital, Shanghai Jiao Tong University School of Medicine, College of Stomatology, Shanghai Jiao Tong University, National Center for Stomatology, National Clinical Research Center for Oral Diseases, Shanghai Key Laboratory of Stomatology, Shanghai Research Institute of Stomatology, Research Unit of Oral and Maxillofacial Regenerative Medicine, Chinese Academy of Medical Sciences, Shanghai, China; 2 Department of Periodontology, Shanghai Ninth People's Hospital, Shanghai Jiao Tong University School of Medicine, College of Stomatology, Shanghai Jiao Tong University, National Center for Stomatology, National Clinical Research Center for Oral Diseases, Shanghai Key Laboratory of Stomatology, Shanghai Research Institute of Stomatology, Research Unit of Oral and Maxillofacial Regenerative Medicine, Chinese Academy of Medical Sciences, Shanghai, China

**Keywords:** labial developmental radicular groove, palatogingival groove, guided tissue regeneration, root canal therapy, periodontal–endodontic lesion

## Abstract

**Objective:**

This study aims to present a multidisciplinary, minimally invasive treatment approach for a rare labial–palatal dual developmental groove associated with anterior malocclusion, emphasizing long-term periodontal stability, functional rehabilitation, and esthetic outcomes.

**Case presentation:**

A 29-year-old female patient presented with a deep labial groove and a shallow distopalatal groove on the maxillary central incisor, complicated by severe localized periodontal destruction and malocclusion. Cone-beam computed tomography (CBCT) revealed a communication between the labial groove and the periapical lesion, whereas the palatal groove was isolated. A comprehensive three-phase treatment protocol was implemented, including root canal therapy combined with apical surgery, guided tissue regeneration (GTR) using Bio-Oss and Bio-Gide, and anterior esthetic rehabilitation through digital smile design and full-ceramic restorations.

**Results:**

After 4 years of follow-up, the probing depth improved from 10 to 1 mm, with radiographic and CBCT evaluations confirming complete bone regeneration and reattachment of periodontal tissues. Functional and esthetic outcomes remained stable, and no recurrence or inflammation was detected during maintenance visits.

**Conclusion:**

This case highlights the diagnostic value of CBCT imaging and the effectiveness of an evidence-based multidisciplinary approach in managing complex labial–palatal dual-groove anomalies. Early diagnosis, bio-ceramic sealing, regenerative periodontal therapy, and meticulous long-term maintenance are essential for achieving predictable and durable clinical success in developmental radicular groove-associated lesions.

## Introduction

Palatogingival groove (PGG), also known as palatal vertical groove, is a developmental anomaly originating near the cingulum of the tooth and extending apically from the cementoenamel junction (CEJ) to varying lengths and depths (1–3). PGG primarily affects maxillary incisors, especially maxillary lateral incisors, with a reported prevalence rate of 0.93%–44.6% across populations, suggesting possible ethnicity-specific variation (1–3).

The etiology of PGG has not been well investigated. Current evidence indicates that inward folding of the inner enamel epithelium and Hertwig's epithelial root sheath during tooth development may result in irregular surface depressions or grooves. These grooves serve as plaque-retentive niches, predisposing affected teeth to localized periodontal and periapical destruction (4, 5).

Several classification systems have been proposed to improve understanding of PGG in both clinical and research contexts (6–9). When the groove occurs on the labial surface, it is termed a labial developmental radicular groove (LDRG) or labial cervical vertical groove. The prevalence of LDRG is notably lower, ranging between 3% and 6.5%, and remains poorly characterized in the literature (10). To define this uncommon developmental anomaly more precisely, we proposed the term “labial–palatogingival groove” (L-PGG) as a unique subtype within the broader PGG classification framework described by Gu (6). The labial developmental radicular groove (L-PGG) is a rare but clinically significant anomaly that occurs on the labial surface of anterior teeth. Its location in the esthetic zone makes it readily visible, often leading to gingival asymmetry, mucogingival recession, and compromised facial esthetics. Biologically, L-PGG acts as a plaque-retentive niche similar to palatal grooves, facilitating bacterial accumulation and forming a direct pathway for periodontal pocket formation and pulpal infection. The thin labial cortical plate further predisposes these sites to rapid inflammatory bone loss and fenestration (11).

To date, despite numerous case reports, no standardized treatment protocol has been established for PGG, and its management remains challenging (5, 12–15). In advanced cases, particularly those involving Gu's Type III grooves with periodontal–endodontic communication, a multidisciplinary approach combining endodontic, periodontal, and restorative therapies is recommended.

This report presents a rare labial–palatal dual developmental groove associated with anterior malocclusion, treated using an integrated multidisciplinary approach involving endodontic, periodontal, and prosthetic procedures.

## Case report

This case report has been written according to Preferred Reporting Items for Case Reports in Endodontics (PRICE) 2020 guidelines (16, 17) (Supplementary Figure S1).

### Informed consent

The patient verbally agreed to undergo the treatment and formally signed an informed consent document authorizing the use of her medical data and subsequent analysis for this case report.

### Preoperative examination

#### Chief complaint

Discomfort while chewing in the left upper tooth for over 1 year.

#### History of present illness

A 29-year-old female product manager presented to the outpatient clinic with a chief complaint of discomfort while chewing in the left upper tooth for over 1 year. After retrieving the history of the present illness, the patient recalled that soreness in the left upper tooth while chewing began 1 year ago, occasionally accompanied by sharp, needle-like pain. She reported no sensitivity to hot or cold stimuli and no history of dental trauma. The patient sought treatment for the remaining teeth while also expressing a desire to improve the esthetic appearance of the anterior teeth. A periodontal cleaning had been performed at another hospital 1 month prior.

### Clinical findings

The patient presented with moderate smile line aesthetics, thin gingival tissue with a high scalloped contour, and an acceptable oral hygiene status. A notable deep overbite and overjet were observed in the anterior region. Detailed examination revealed that Tooth #21 exhibited abnormal crown morphology with discoloration, a developmental groove deformity on the labial cervical area with a probing depth (PD) of 10 mm and positive bleeding on probing (BOP), and a developmental groove deformity on the palatal distal cervical area with a PD of 2 mm and negative BOP. Tooth #21 also showed no gingival erythema or swelling and no percussion sensitivity or mobility after testing. Apart from Tooth #21, Teeth #11 and #12 showed smooth, white striated lesions on the labial crown surfaces. A full-mouth periodontal examination noted a mean probing depth of 1.2 mm, and clinical attachment loss was observed only in Tooth #21 (10 mm), with no loss detected in other teeth.

**Figure 1:**
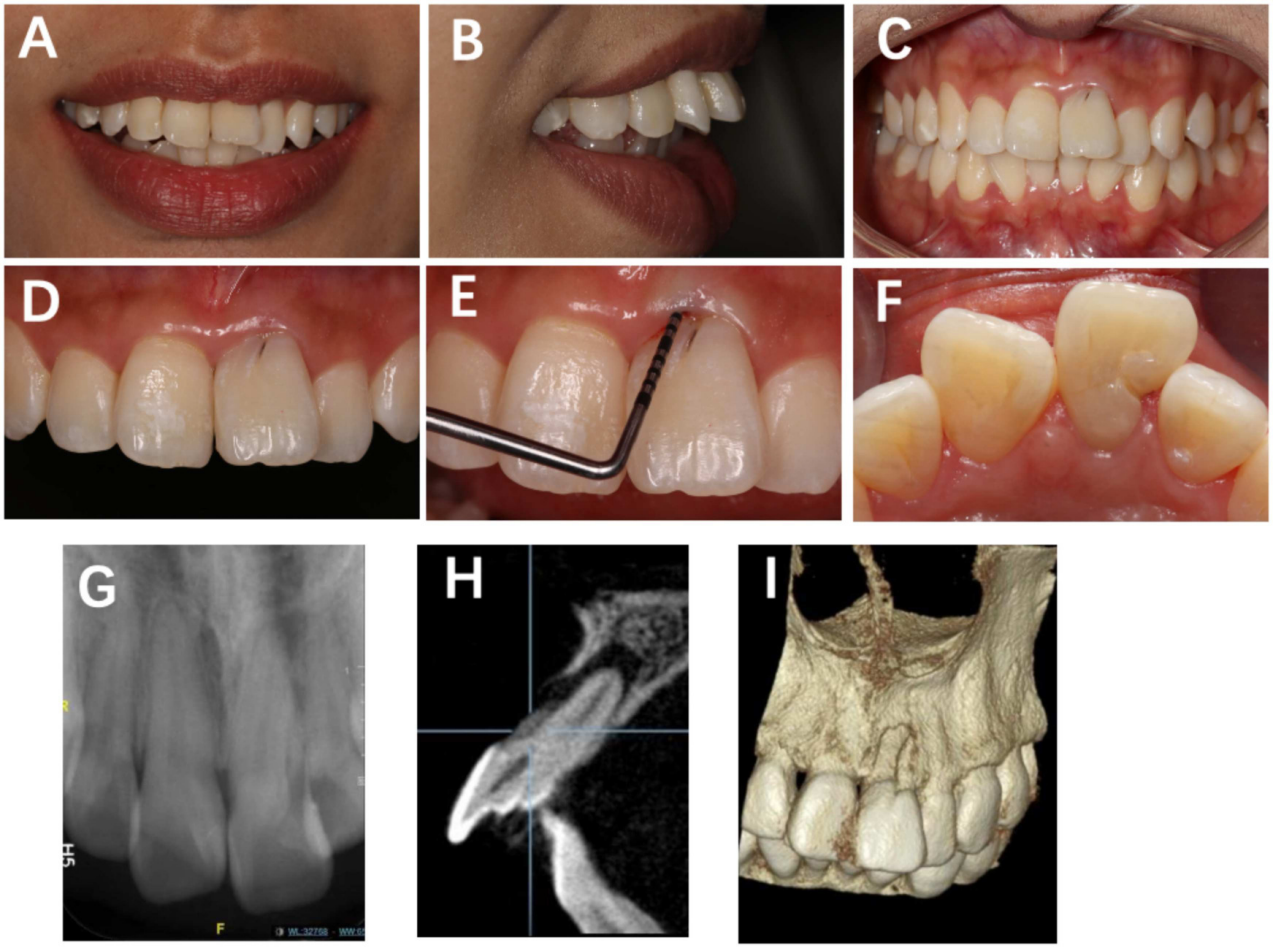
Preoperative clinical view of Tooth #21 showing a distinct labial developmental groove extending from the cingulum toward the gingival margin, accompanied by localized periodontal pocketing. **(A)** Frontal smile view; **(B)** lateral smile view; **(C)** frontal view in centric occlusion; **(D)** labial surface of Tooth #21 showing a developmental groove; **(E)** probing depth of 10 mm at the mid-labial site; **(F)** shallow depression (2 mm) on the distolingual marginal ridge; **(G)** periapical radiograph showing two root canals, one poorly visualized; **(H)** periapical radiolucency surrounding the root apex; **(I)** CBCT 3D reconstruction demonstrating a labial groove along the root surface.

Subsequent periapical radiograph (Dentsply Sirona, Ballaigues, Switzerland) revealed a bifid root canal configuration in Tooth #21 with indistinct morphology, accompanied by a periapical radiolucent area (Figure 1). Cone-beam computed tomography (CBCT) (KaVo Kerr, Brea, CA, USA) 3D reconstruction demonstrated that a developmental groove on the labial root surface of Tooth #21 extends to the apex, with associated labial bone loss. A palatal groove can also be observed (Figure 1F).

### Diagnosis and treatment plan

The diagnosis was Type III LDRG and Type I PGG (according to Gu's classification) ﻿with a combined periodontal–endodontic lesion in Tooth #21. Except for the main diagnosis, several other ones have also to be underlined: crowded dentition accompanied by deep overbite and overjet, mild dental fluorosis in Teeth #11 and #12 (Dean's classification), and chronic gingivitis.

After a comprehensive evaluation of the patient's overall oral condition and based on general dentistry principles, two critical issues require focused discussion for this case:

1. Preservation viability of the developmental groove on Tooth #21
2. Esthetic rehabilitation design for the anterior region

Two treatment approaches were designed and generated accordingly then: detailed strategies were listed in the Supplement Material. Since the palatal PGG is classified as Type I and does not extend subgingivally, this case report primarily focuses on the relevant treatment strategies for LDRG.

After thorough consultation, the patient explicitly expressed a preference for preserving Tooth #21 and declined orthodontic intervention because of its extended duration and high costs. Between tooth extraction/implant and preservation strategies, the patient ultimately selected Option 1B (Supplementary Figure S2). Therefore, the treatment will be implemented in two phases: (1) combined endodontic–periodontal therapy and developmental groove management for Tooth #21 and (2) full-crown and veneer restorations for anterior teeth (#12–22). The entire process will incorporate oral hygiene education, periodontal maintenance, and scheduled follow-ups (see Supplementary Figure S3 for the treatment flowchart). Notably, prior to definitive restoration, potential occlusal risks were evaluated considering the patient's deep overbite and mild anterior crowding. The absence of orthodontic correction could predispose the restored tooth (#21) to increased functional load during protrusive and incisal guidance.

### Treatment sequence

#### Tooth 21’s non-surgical and surgical treatment

**Figure 2:**
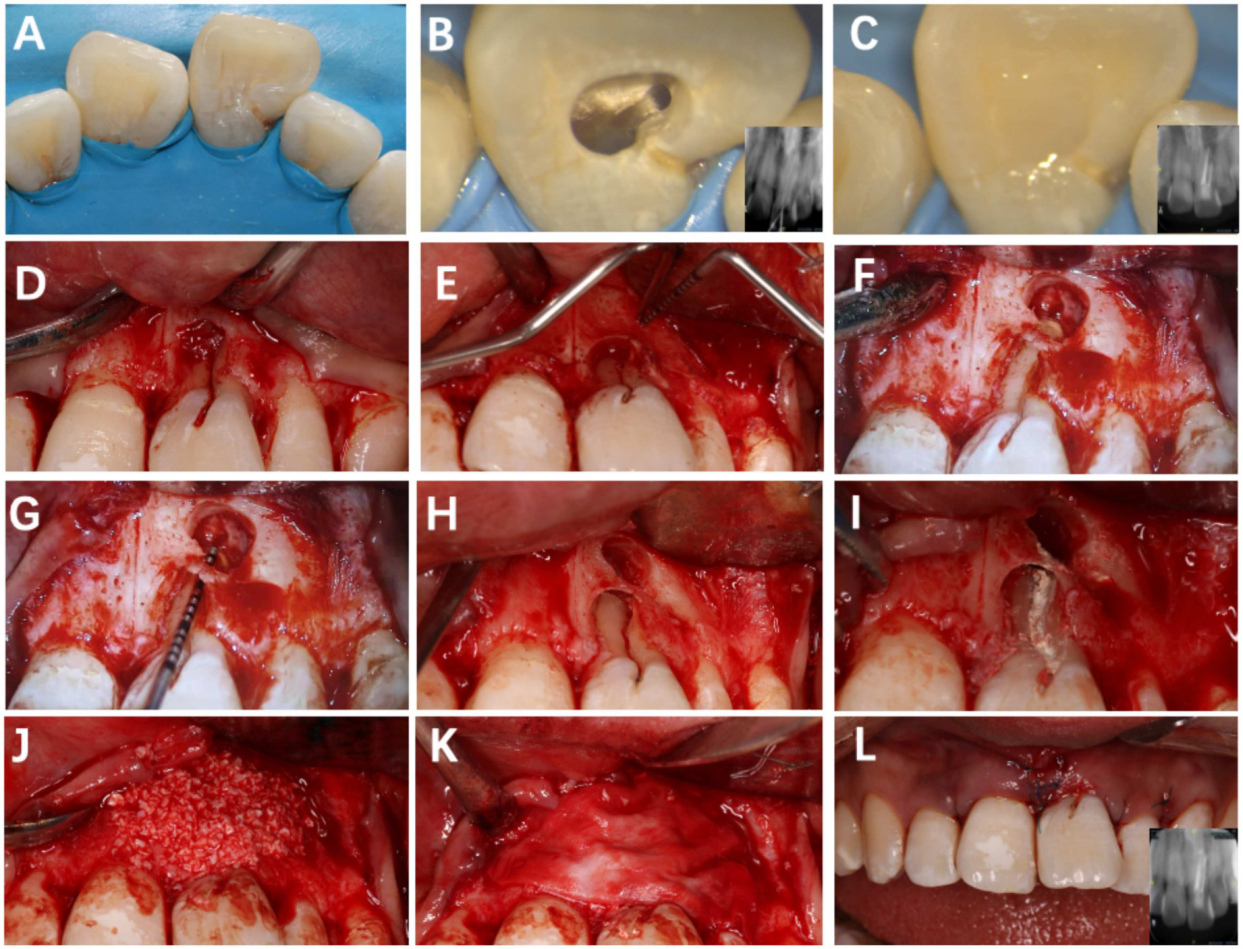
Root canal treatment and flap surgery of tooth #21. **(A)** Rubber dam isolation; **(B)** observation of two canal orifices; **(C)** immediate postoperative lingual surface photograph; **(D)** papilla-sparing flap elevation; **(E)** exposure of the lesion area; **(F)** debridement of granulation tissue; **(G)** determination of root length; **(H)** root-end resection, retro-preparation, and retrograde filling; **(I)** sealing of the labial groove with iRoot BP Plus; **(J,K)** placement of Bio-Oss graft and Bio-Gide membrane; **(L)** sutured flap.

The treatment process for Tooth #12 consisted of two phases:

1. Root canal treatment phase (Figures 2A–C): A non-surgical root canal therapy was performed on Tooth #21 under rubber dam isolation. Upon opening the pulp chamber, a mesiodistal variation in the root canal orifice was observed. Further exploration confirmed a double-canal morphology, verified by inserting gutta–percha points and taking periapical radiographs. After completing the root canal preparation, obturation was performed using iRoot SP bio-ceramic sealer with the single-cone technique. The coronal restoration was completed with composite resin. Figure 2C shows the immediate postoperative root filling effect.
2. Apical surgery phase (1 month later) (Figures 2D–L): After local anesthesia, a full-thickness mucoperiosteal flap was gently elevated on the labial aspect of Tooth #21 to expose the defect. The developmental groove was cleaned, smoothened with a fine diamond bur, and sealed with iRoot BP Plus to ensure a tight barrier against bacterial penetration. Apical root-end resection and retrograde filling were performed. The bony defect was then filled with Bio-Oss particulate bone graft, and a Bio-Gide membrane was carefully adapted to cover the area before primary closure with interrupted 6-0 sutures. Postoperative antibiotics and chlorhexidine rinses were prescribed for 1 week.

No intraoperative complications occurred. However, the procedure presented several technical challenges: The labial cortical plate was thin and irregular, limiting surgical access and membrane adaptation. In addition, achieving a stable flap position required meticulous handling to avoid tearing the delicate soft tissue. These issues were managed through atraumatic flap reflection, incremental graft packing, and tension-free suturing.

### Anterior area direct resin restoration

**Figure 3:**
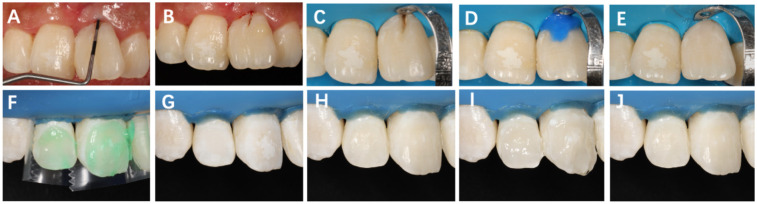
Restorative procedure following surgery on Tooth #21. **(A)** One month postoperatively, probing depth reduced to 3 mm; **(B)** intraoral shade selection using composite resin; **(C)** rubber dam isolation and cavity preparation after caries removal; **(D)** acid etching and adhesive application; **(E)** restoration with flowable resin. **(F)** Surface conditioning with 15% hydrochloric acid gel for 3 min; **(G)** rinsing and air drying; **(H)** application of anhydrous ethanol for 30 s; **(I)** infiltrating resin with oxygen barrier and light-curing for 40 s; **(J)** final polishing of the restoration surface.

The 1-month postoperative follow-up examination revealed that the gingival color, contour, and texture had largely returned to normal, with probing depth improving from the preoperative measurement of 10 to 3 mm. Subsequently, caries treatment and resin composite restoration were performed on the developmental groove of the crown of Tooth #21 (Figure 3). Additionally, the resin infiltration technique was applied to the labial surfaces of Teeth #11 and #12 for provisional esthetic enhancement to improve their coloration.

### Anterior area indirect aesthetic recovery period

**Figure 4:**
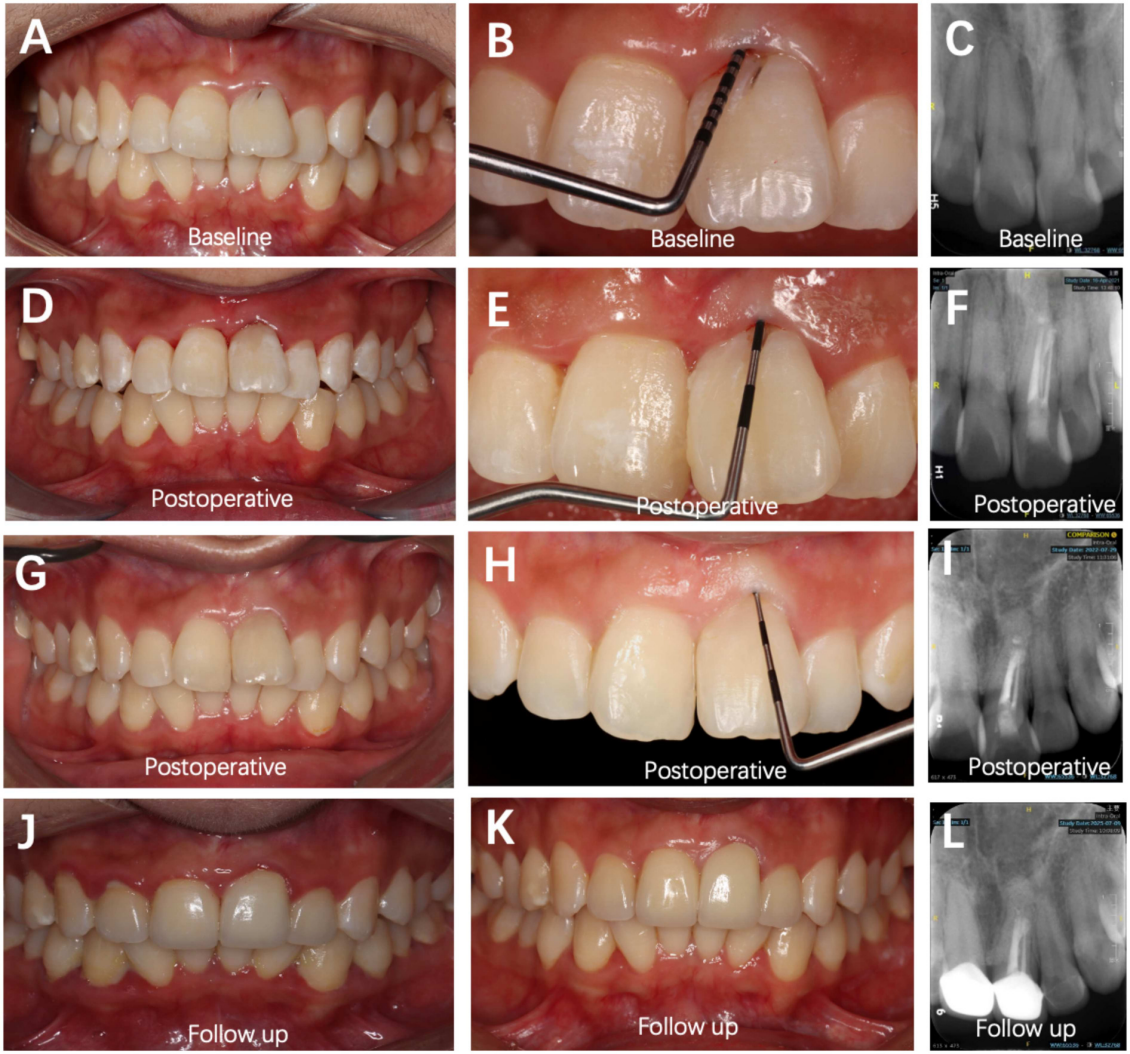
Sequential intraoral and radiographic evaluations of Tooth #21 at different time points (baseline, postoperative, and follow-up). **(A–C)** Preoperative images showing a PD of 10 mm at the labial site. **(D–F)** Postoperative views demonstrating soft-tissue healing with a PD of 3 mm. **(G–I)** Fifteen months postoperatively, PD remained 1 mm, and increased bone density was evident on the labial aspect of the root. **(J,L)** Fifty months postoperatively, clinical and radiographic evaluations revealed a stable periodontal condition, with no signs of root resorption or periapical inflammation. **(K)** Fifty-five months postoperatively, clinical image showing stable periodontal status (PD = 2 mm).

At the 15-month postoperative follow-up, Tooth #21 exhibited a stable periodontal PD of approximately 1 mm, with normal gingival color, contour, and texture. Radiographic examination revealed favorable bone formation in both the periapical region and the labial grafted area of Tooth #21 (Figure 4).

The restoration procedure primarily involves the following key steps: First, digital smile design (DSD) planning was conducted preoperatively, followed by the fabrication of diagnostic wax-ups and silicone guides. Next, tooth preparation was performed, and temporary restorations were created and tried on. Finally, the permanent prosthesis was fabricated and clinically bonded into the natural teeth (Supplementary Figure S4). This entire process established a comprehensive digital restoration workflow.

### Follow-up

The 4-year follow-up examination revealed that the patient's periodontal health remained in good condition (Figure 4). Periapical radiographs and CBCT scans demonstrated stable healing in the original apical region and buccal bone tissue. The aesthetic restoration of the anterior teeth achieved satisfactory results, with significantly improved occlusal function compared with pre-treatment status.

## Discussion

Developmental grooves, particularly palatogingival grooves (PGGs), are morphological anomalies predisposing maxillary incisors to combined endodontic–periodontal lesions (2, 3, 5, 18). While most PGGs are located on the palatal aspect, the present case exhibited an unusual dual-groove morphology, characterized by a deep labial groove accompanied by a shallow distopalatal groove. This variant implies a complex radicular invagination extending across both developmental planes, rarely documented in clinical literature.

CBCT was instrumental in clarifying the 3D morphology and identifying the communication between the labial groove and the periapical lesion (2, 9, 19). The palatal groove was shallow and isolated, showing no pulpal involvement. This dual-groove configuration complicated diagnosis, requiring differentiation between primary endodontic and secondary periodontal lesions. Such imaging evidence emphasizes the diagnostic and prognostic value of CBCT in assessing radicular anomalies.

According to Gu's classification (Supplementary Figure S5), this case corresponds to a Type II–III transitional morphology, where the groove extends beyond the cementoenamel junction but remains short of complete root separation (6). Understanding this anatomical category was crucial in designing a minimally invasive surgical protocol and predicting regenerative outcomes.

The case was successfully managed through a multidisciplinary approach involving root canal therapy (RCT), apical surgery, guided tissue regeneration (GTR), and prosthetic rehabilitation (Supplementary Figure S5). The groove was sealed using iRoot BP Plus, a bio-ceramic material with excellent sealing ability, bioactivity, and antibacterial properties (20, 21). Owing to its osteo-inductive characteristics, it is particularly suited for root-end obturation. However, given its limited esthetics and lack of chemical adhesion, a two-stage restorative protocol was applied, using composite resin coronally for enhanced esthetic and functional integration as recommended by Yan et al. (22).

Periodontal health maintenance was identified as a key determinant of long-term prognosis. Objective parameters were added to this report: The plaque index decreased from 27% to 14%, and bleeding on probing reduced from 16% to 4% following initial therapy (Supplementary Figure S6). The patient, a non-smoker, maintained excellent oral hygiene, aligning with previous studies demonstrating that plaque control and smoking cessation significantly influence PGG treatment success (5).

Recent evidence has reaffirmed the value of CBCT in PGG management. A 2025 systematic review and meta-analysis confirmed that CBCT surpasses 2D imaging in detecting subtle labial and palatal grooves and quantifying their prevalence (19). Large-scale cohort studies (*n* ≈ 900) demonstrated correlations between groove morphology, periapical pathology, and periodontal bone loss (2). Moreover, CBCT-based nomograms and risk prediction models have been proposed for preoperative assessment of radicular grooves (3, 5, 23). These findings underscore the expanding diagnostic and research potential of CBCT in complex groove-associated lesions.

Following periodontal stabilization, esthetic restoration was completed using DSD, diagnostic wax-up, and silicone guides. This digital workflow optimized the final esthetics and occlusal harmony without orthodontic intervention (24). Combined veneer and full-ceramic crowns achieved natural esthetics and stable occlusion, verified during a 3-year follow-up.

Despite favorable outcomes, several limitations exist. First, a mild crown–root ratio discrepancy between Tooth #21 and its contralateral Tooth #11 was noted, potentially improvable by periodontal recontouring. Second, although 4-year follow-up images showed complete osseous repair and stable function, mild gingival erythema persisted (Figure 4J). It is noteworthy that the latest follow-up imaging (Figure 4K) after completing periodontal initial therapy shows significant improvement in periodontal health compared with the previous examination(Figure 4J). This result once again confirms the importance of long-term periodontal maintenance throughout the entire life cycle of teeth. Finally, during apical surgery, the coronal segment of the groove was not sealed to minimize contamination risk; this was later completed under rubber dam isolation. Objective periodontal indices will continue to be monitored to assess long-term stability.

Looking ahead, future research should focus on developing standardized diagnostic and treatment algorithms for labial–distopalatal radicular grooves (LDRGs) (3, 19, 25). Long-term prognosis could be better evaluated using quantitative digital imaging and artificial intelligence (AI)-assisted CBCT analysis to measure bone density and tissue remodeling dynamically (26).

In conclusion, this case highlights the diagnostic challenges and treatment considerations of a rare labial–palatal dual-groove variant. Integration of CBCT imaging, bio-ceramic sealing, and periodontal regeneration within a minimally invasive multidisciplinary framework yielded predictable long-term esthetic and functional success. This case underscores the need for precise imaging, individualized treatment planning, and strict periodontal maintenance in managing complex PGG-related lesions.

## Conclusion

This case demonstrates that precise CBCT-based diagnosis, multidisciplinary minimally invasive management, and careful periodontal maintenance can achieve long-term functional and esthetic success in teeth affected by complex dual-groove morphology. Beyond the individual outcome, this report provides clinical evidence supporting an evidence-based, integrated approach for managing rare radicular groove anomalies.

The treatment protocol described herein may serve as a reference for clinicians when dealing with similar endodontic–periodontal lesions of developmental origin, emphasizing the importance of early detection, comprehensive imaging evaluation, and interdisciplinary collaboration to ensure predictable outcomes.

## Data Availability

The original contributions presented in the study are included in the article/Supplementary Material, further inquiries can be directed to the corresponding author.
