# Supplementary material for: Case Report: Multidisciplinary management of a rare labial–palatal dual developmental groove with malocclusion: a 4-year follow-up
Source: Front Oral Health. 2025 Nov 20;6:1705402. doi: 10.3389/froh.2025.1705402 (PMC12675441; doi:10.3389/froh.2025.1705402)
Supplement: Supplementary file 1 [file Table1.docx]

**Supplementary Figures**


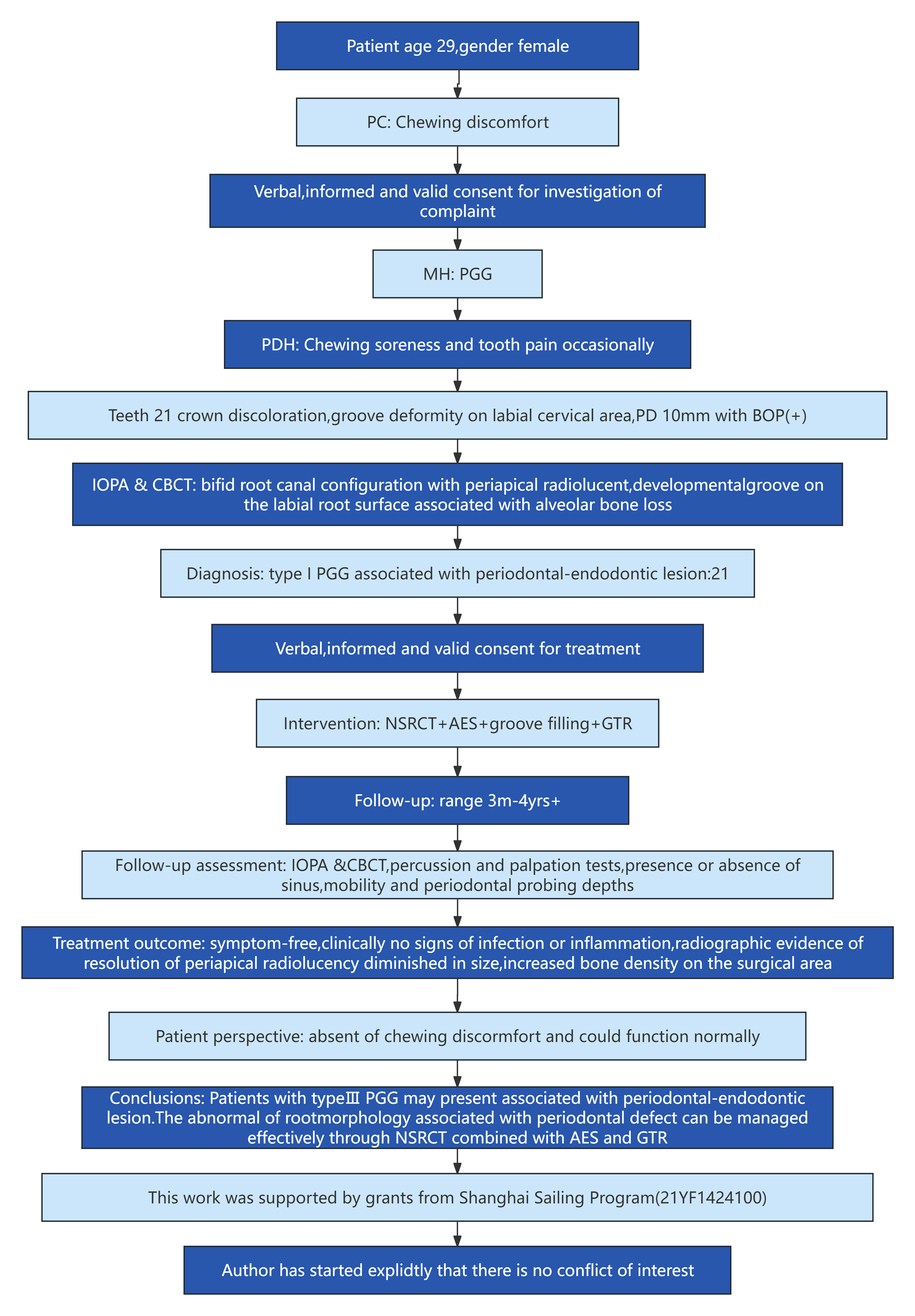


**FIGURE S1**. PRICE 2020 flow chart


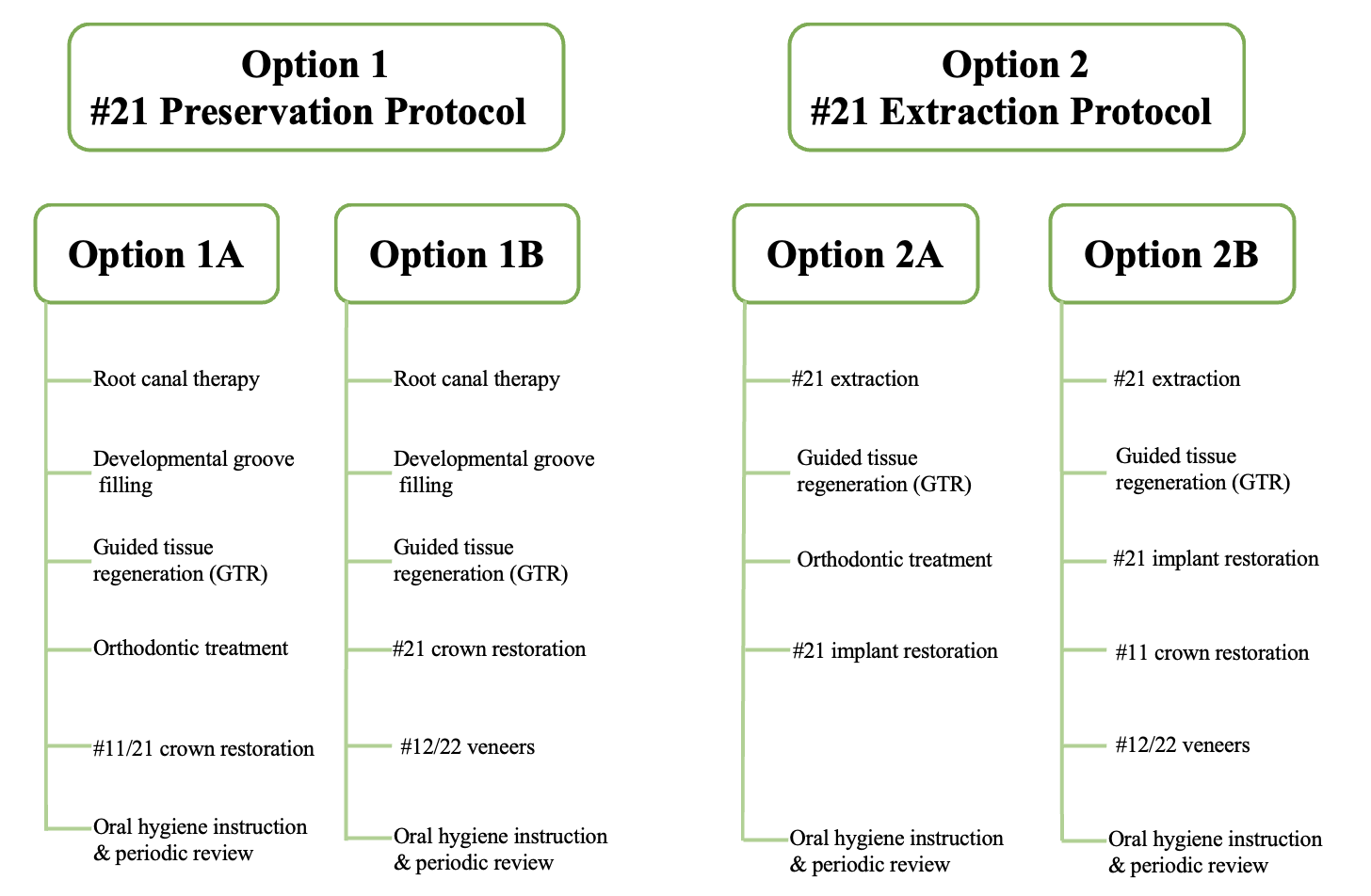


**FIGURE S2**. Multidisciplinary Treatment Options for Tooth #21: Preservation vs. Extraction Protocols


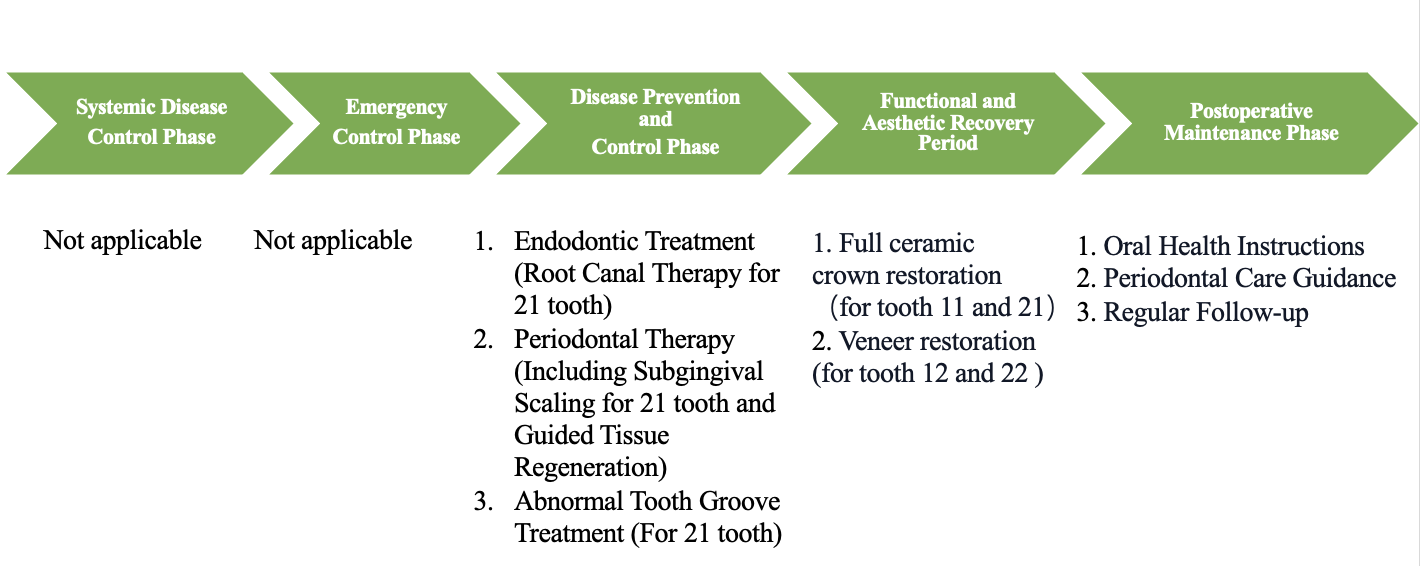


**FIGURE S3.** Multidisciplinary Treatment Flowchart for Tooth #21 with Labial–Palatal Dual Developmental Groove and Malocclusion


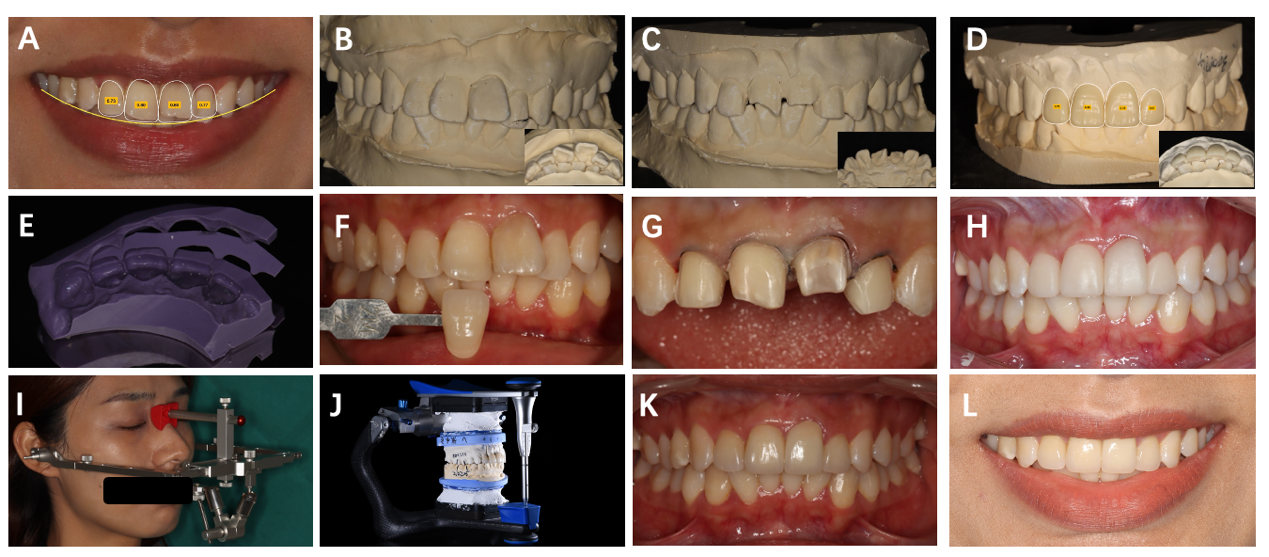


**FIGURE S4.** (A) Digital Smile Design (DSD) design; (B) Study model; (C) Tooth preparation on study model; (D) Wax-up based on DSD design; (E) Silicone template for tooth preparation; (F) Shade selection; (G) Guided tooth preparation; (H) Provisional restoration try-in; (I) Face-bow transfer: (J) Mounting the casts on the articulator; (K) Immediate image after restorations cementation; (L) Smile record


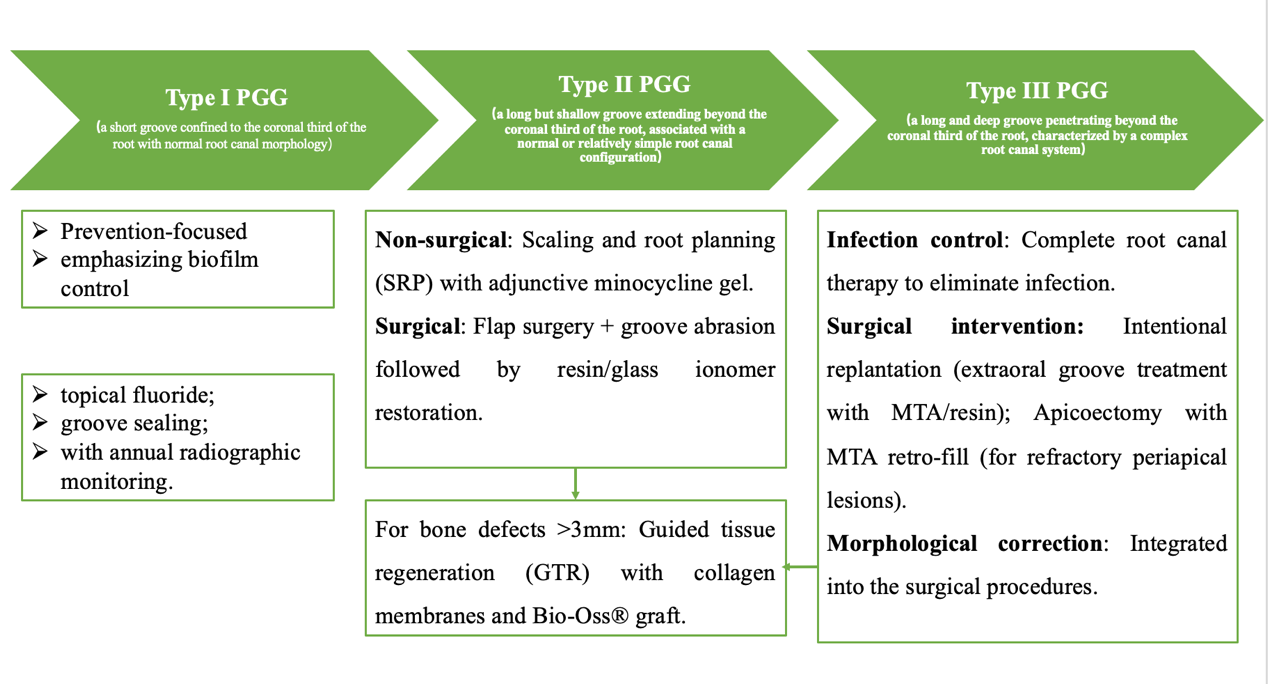


**FIGURE S5.** Clinical Strategies for Three Types of PGG


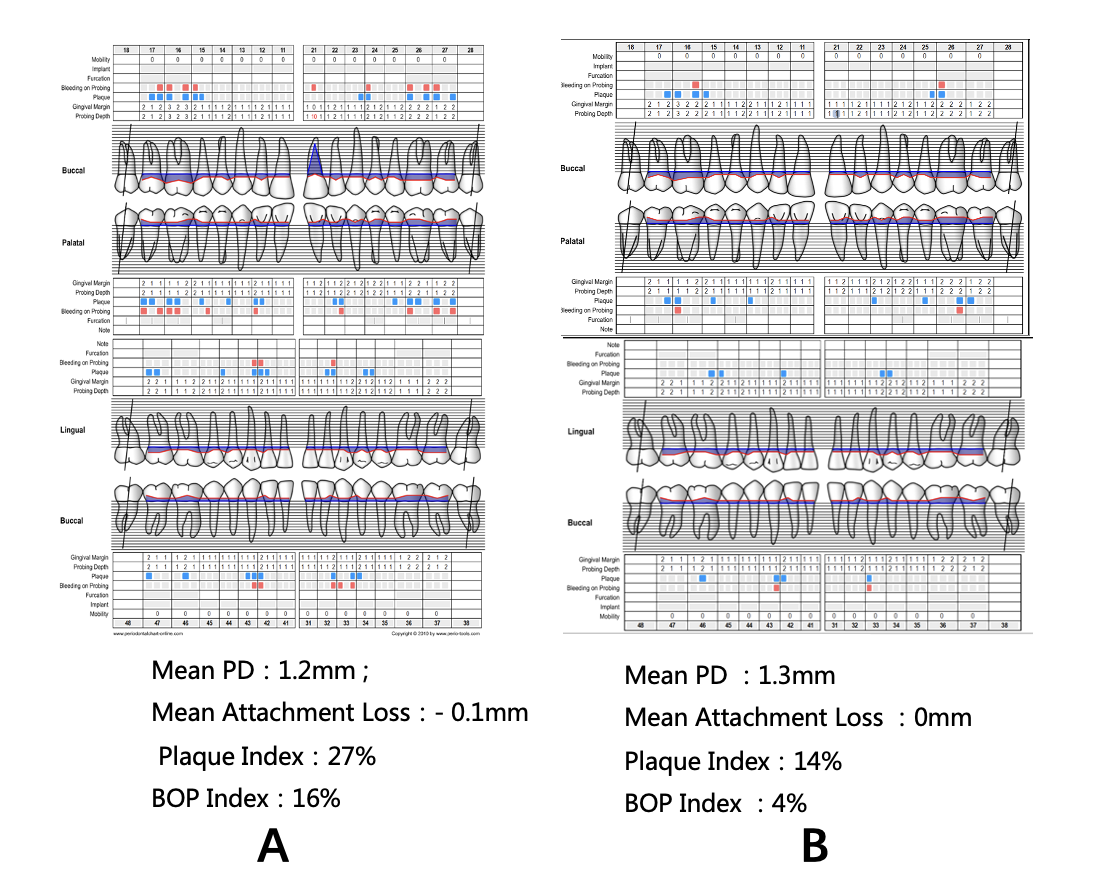


**FIGURE S6.** Comparative Analysis of Periodontal Parameters in Patients Before Surgery（A） and at 15 Months Postoperatively（B）
